# Agreement of thermodilution and direct Fick methods for cardiac output across varying haemodynamic conditions

**DOI:** 10.1093/eschf/xvaf001

**Published:** 2026-01-08

**Authors:** Michael Melin, Ida Haugen Löfman, Dmitri Matan, Magnus Nygren, Bahira Shahim, Lars H Lund, Aristomenis Manouras

**Affiliations:** Heart and Vascular and Neuro Theme, Division of Cardiology, Karolinska University Hospital, Stockholm, Sweden; Department of Laboratory Medicine, Huddinge, Karolinska Institutet, Stockholm, Sweden; Heart and Vascular and Neuro Theme, Division of Cardiology, Karolinska University Hospital, Stockholm, Sweden; Department of Medicine, Solna, Karolinska Institutet, Stockholm, Sweden; Heart and Vascular and Neuro Theme, Division of Cardiology, Karolinska University Hospital, Stockholm, Sweden; Department of Medicine, Huddinge, Karolinska Insitutet, Stockholm, Sweden; Heart and Vascular and Neuro Theme, Division of Cardiology, Karolinska University Hospital, Stockholm, Sweden; Heart and Vascular and Neuro Theme, Division of Cardiology, Karolinska University Hospital, Stockholm, Sweden; Department of Medicine, Solna, Karolinska Institutet, Stockholm, Sweden; Heart and Vascular and Neuro Theme, Division of Cardiology, Karolinska University Hospital, Stockholm, Sweden; Department of Medicine, Solna, Karolinska Institutet, Stockholm, Sweden; Heart and Vascular and Neuro Theme, Division of Cardiology, Karolinska University Hospital, Stockholm, Sweden; Department of Medicine, Solna, Karolinska Institutet, Stockholm, Sweden

**Keywords:** Thermodilution, Fick, Cardiac output, Tricuspid regurgitation, Pulmonary hypertension, Pulmonary vascular resistance

## Abstract

**Introduction:**

The direct Fick method is the gold standard for measuring cardiac output (*Q*). However, thermodilution (TD) is more widely used in clinical practice due to its feasibility. As the accuracy of TD-derived *Q* (*Q*_TD_) may vary by flow state, it can influence the estimation of pulmonary vascular resistance (PVR) and pulmonary hypertension (PH) classification. This study aimed to compare *Q*_TD_ with Fick-derived *Q* (*Q*_F_) across haemodynamic states and evaluate the clinical reliability of TD-derived PVR (PVR_TD_).

**Methods:**

We retrospectively analysed 852 right heart catheterizations (2014–22) with simultaneous *Q*_TD_ and *Q*_F_ measurements.

**Results:**

64% of patients had heart failure, and 20% had pulmonary arterial hypertension. *Q*_TD_ correlated strongly with *Q*_F_ (*r* = 0.78, *P* < .001) but demonstrated a flow-dependent bias—overestimating *Q* in low-flow states (+0.1 L/min) and underestimating it in normal/high flow (−0.48 L/min). Despite this, PVR_TD_ accurately identified elevated PVR_F_ at both 2 and 5 Wood unit thresholds (area under the curve = 0.97, *P* < .001). Agreement between *Q*_TD_ and *Q*_F_ was unaffected by tricuspid regurgitation severity.

**Conclusion:**

Although TD shows a predictable bias across flow states, it remains a clinically reliable method for estimating elevated PVR. These findings support the clinical utility of TD in PH assessment, while emphasizing the need for direct Fick measurement in low-output states—particularly in patients evaluated for advanced therapies such as transplantation.

## Introduction

Cardiac output (*Q*) is a pivotal haemodynamic parameter with significant diagnostic and prognostic implications. It is integral to the characterization of heart failure (HF) and pulmonary hypertension (PH), and is crucial for guiding pharmacological management, decision-making for mechanical circulatory support, and assessing eligibility for heart transplantation.^1–3^ The direct Fick method is considered the gold standard for *Q* measurement. While it provides essential haemodynamic data and serves as the reference for validating alternative *Q* methods, its dependence on precise VO_2_ measurement and mixed venous sampling limits routine applicability.

Thermodilution (TD), introduced in 1971, offers a more accessible alternative. It employs a pulmonary artery catheter with a distal thermistor to measure changes in blood temperature after injection of a known fluid volume, providing a practical and widely adopted method for *Q* assessment^4^ Multiple studies have shown strong correlation and agreement between TD and Fick, supporting TD’s clinical utility.^5–11^

Nevertheless, discrepancies between the two methods have been reported, particularly under specific haemodynamic conditions.^11–16^ Thermodilution is traditionally considered less reliable in the presence of significant tricuspid regurgitation (TR), where regurgitant flow may distort thermal signal and lead to *Q* overestimation.^16,17^ Recent evidence, however, question this limitation.^5,18^ The consistency of TD–Fick agreement across flow states also remains unclear, with conflicting evidence regarding *Q* overestimation by TD in low-output settings.^10,11,14^ Importantly, the limited scope of these studies—often conducted on selected patient populations and relatively small sample sizes—limits their generalizability.^13^ Accurate *Q* estimation is particularly important for calculating pulmonary vascular resistance (PVR), a flow-dependent metric central to PH classification. Whether discrepancies between TD and Fick compromise the reliability of TD-derived PVR and thus impact PH diagnosis has not been systematically evaluated.

The objective of this study was to assess the diagnostic accuracy and clinical utility of TD-derived PVR, using Fick-derived PVR as the reference standard and to evaluate agreement between TD- and Fick-derived *Q* across varying flow states.

## Methods

### Study population

All consecutive right heart catheterization (RHC) at Karolinska University Hospital from February 2014 to December 2023 (*n* = 1062) were included. Indications for RHC included unclear dyspnoea, HF assessment, constrictive pericarditis evaluation, and investigation of PH. Patients with intracardiac shunts, acute coronary syndrome (within 3 months), or recent cardiac surgery (within 1 year) were excluded. Per clinical protocol, all patients were afebrile and free of active infection. The study adhered to the Declaration of Helsinki and received ethics approval from the local ethics committee (nr 2024-02861-01). Patient consent was not required for this retrospective review of patient records.

### Echocardiography

All patients underwent transthoracic echocardiography (E9 system, GE Ultrasound, Norway) with a 2.5 MHz transducer, following current guidelines. Three-cycle 2D images were obtained and analysed offline (EchoPAC, GE Ultrasound) by a blinded sonographer. Left ventricular end-diastolic and end-systolic volumes and ejection fraction (EF) were measured via the Simpsons biplane method. Valvular heart disease was graded as mild, moderate, severe, massive, and torrential, per recommendations. For the purposes of the study, the severe, massive, and torrential TR comprised together the group of severe TR denoted as TR-3, whereas mild and moderate TR are denoted as TR-1 and TR-2, respectively. For patients with greater than moderate TR, severity was assessed using 3D vena contracta area (3D-VCA) and categorized as severe (effective regurgitant orifice 0.75–0.94 cm^2^), massive (0.95–1.14 cm^2^), or torrential (≥1.15 cm^2^).^19^

### Right heart catheterization

Right heart catheterization was performed within 1 h of echocardiography. A flow-guided pulmonary artery catheter was placed percutaneously guided by ultrasound (Vivid E9, GE Healthcare Co., Milwaukee, WI, USA) under local anaesthesia (Carbocain 20 mg/mL, Aspen Nordic, Denmark). Using a double Seldinger technique, access to the internal jugular vein was created via an 18 G needle (Cordis, Miami Lakes, FL, USA), followed by insertion of a guidewire. After vein placement was confirmed, an introducer sheath (7 Fr, St Jude/Abbott Medical, Plymouth, MN, USA) was slid over the wire, which was then removed. Finally, a Swan–Ganz balloon-tipped catheter (7 Fr, Edwards Lifescience, Irvine, CA, USA) was inserted and advanced through the right atrium, right ventricle, and pulmonary artery in a wedge position before being returned to the pulmonary artery. Combined fluoroscopy (OEC Fluorostar 7900, GE Healthcare Co., Milwaukee, WI, USA) and pressure wave morphology was used to confirm correct catheter placement.

Mean right atrial pressure (RAP), systolic (PAPs), diastolic (PAPd), mean pulmonary artery pressure (PAPm), and mean pulmonary artery wedge pressure (PAWPm) were recorded under fluoroscopy after calibration, with the zero-level set at the mid-thoracic line. Pressure tracings were stored (Sensis Vibe Hemo, Siemens Healthineers USA) and analysed offline using the post-processing workstation (Sensis Vibe Post Processing Workstation, Siemens Healthineers USA). PAWPm measurements were averaged from a minimum of five heart cycles at spontaneous end-expiration. Blood gases were analysed using ABL90 FLEX. *Q* was assessed with direct Fick and TD as described below. Pulmonary vascular resistance was obtained as per the equation PAPm–PAWPm/*Q* separately for TD and Fick-derived *Q* measurements. Left heart disease associated PH (PH-LHD) was subsequently defined as PAPm >20 mmHg and PAWPm ≥15 mmHg at rest or PAWPm ≥25 mmHg during exercise. Pulmonary arterial hypertension (PAH) was finally defined as PAPm >20 mmHg and PAWPm 3 WU.

### Direct Fick’s method

In each patient, a sample of exhaled air was collected by firmly applying a mask over the patient’s face and nose, until steady state was reached. Oxygen consumption (VO_2_) was then analysed using breath-by-breath expired gas analysis at a steady state, utilizing a metabolic cart (Vyntus CPX with a Rudolph breathing mask, model 7450). Calibration was performed prior to each measurement.

The systemic arterial and mixed venous samples were drawn from the radial and the pulmonary artery, respectively, into a 5 mL syringes and analysed immediately with an oximeter (Xpod3012LP, Nonin). At the same time, haemoglobin concentration was measured. Arterial–venous oxygen content difference (AVO_2_-diff) was measured directly as the [difference between systemic and pulmonary arterial O_2_ contents as per equation AVO_2_-diff = (SaO_2_ × haemoglobin × 1.34) − (SvO_2_ × haemoglobin × 1.34)].

Direct Fick-derived *Q* (*Q*_F_) was determined as the ratio between VO_2_ and the AVO_2_-diff i.e. *Q*_F_ = VO_2_/AVO_2_-diff. Cardiac index (*Qi*_F_) was calculated as the *Q*_F_ divided by the body surface area (BSA) measured according to the Du Bois formula. The direct Fick method was used as the reference standard for all analyses comparing cardiac output and derived parameters with those obtained by TD.

### Thermodilution

A bolus of 10 mL iced 0.9% NaCl was injected into the RA through the Swan–Ganz catheter. The procedure was repeated –five to seven times at 1 min interval. The analysis was performed using the Siemens Healthineers USA. *Q*-derived by TD (*Q*_TD_) was calculated as the average value of three measurements with <10% difference between each other.

### Biomarkers

N-terminal pro B-type natriuretic peptide was analysed by proBNPII (Roche Diagnostics, Bromma, Sweden). The estimated glomerular filtration rate (eGFR) was calculated according to the equation: eGFR = 175 × [creatinine]−1.154 × −0.203 × 0.742 [if female] mL/min/1.73; creatinine in mg/dL, age in years.

### Statistics

Continuous variables are reported as mean ± standard deviation (SD) or median (interquartile range), based on the Shapiro–Wilk normality testing. Group comparisons employed unpaired *t*-tests or Mann–Whitney *U* tests, as appropriate; categorical variables were compared using the *χ*^2^ or Fisher’s exact tests. The absolute per cent error (APE) between TD and Fick was calculated as |Δ*Q*_TD–F_|/*Q*_F_ × 100. Precision was assessed using typical error and coefficient of variation. Associations between TD and Fick were evaluated using the Spearman correlation with scatter plots; agreement was assessed via the Bland–Altman analysis. Diagnostic performance was evaluated using receiver operating characteristic (ROC) analysis and the area under the curve (AUC), as well as *χ*^2^ tests to assess sensitivity, specificity, positive predictive value (PPV), and negative predictive value (NPV). *Qi*_F_ was categorized as low (≤2.2), normal (2.2–4.1), or high (>4.1 L/min/m^2^). Pulmonary hypertension severity was stratified as non-PH, mild (PAPm 21–30 mmHg), moderate (31–40 mmHg), and severe (>40 mmHg). Linear regression was performed to identify covariates associated with the difference between Δ*Q*_TD–F_. Two-sided *P*-values <.05 were considered statistically significant. Analyses were conducted using SPSS (IBM, NY, USA).

## Results

A total of 1062 RHC examinations were reviewed. Simultaneous *Q* measurements by both the Fick and TD methods were documented in 852 RHCs in individual patients, constituting the study cohort. Demographic data are presented in *Table 1*. The median age was 66 years (54–75); 46% women.

**Table 1:** Demographic data

**Age**	66 (54; 75)
**Female (*n*, %)**	393 (46)
**BSA**	1.92 ± 0.24
**Diagnosis**	
HF (*n*, %)	542 (64)
HFpEF (*n*, %)	309 (36)
HFmrEF (*n*, %)	73 (9)
HFrEF (*n*, %)	160 (19)
PAH (*n*, %)	170 (20)
Constrictive cardiomyopathy (*n*, %)	19 (2.2)
AF (*n*, %)	267 (31)
PM/CRT-D (*n*)	64/127
Hypertension (*n*, %)	407 (48)
Diabetes mellitus (*n*, %)	159 (19)
Hypercholesterolaemia (*n*, %)	145 (17)
Ischaemic heart disease (*n*, %)	164 (19)
NYHA I (*n*, %)	89 (10)
NYHA II (*n*, %)	152 (18)
NYHA III (*n*, %)	564 (66)
NYHA IV (*n*, %)	32 (4)
**Treatment**	
ARB/ACE-I/ARNI (*n*, %)	463 (54)
Loop diuretics (*n*, %)	533 (63)
Beta-blockers (*n*, %)	537 (63)
MRA (*n*, %)	397 (47)
Calcium channel blockers (*n*, %)	124 (15)
**Laboratory findings**	
NT-proBNP (ng/L)	1020 (356; 2350)
Creatinine	91 (72; 112)
Haemoglobin (mg/L)	133 (121; 146)

Demographic characteristics of the study cohort.

HF, heart failure; HFpEF, HF with preserved ejection fraction; HFmrEF, HF with mid-range ejection fraction; HFrEF, HF with reduced ejection fraction; PAH, pulmonary arterial hypertension; AF, atrial fibrillation or flutter; PM, pacemaker; CRT-D, cardiac resynchronization treatment; NYHA, New York Heart Association functional class; ARB, angiotensin receptor blocker; ACE-I, ACE-inhibitor; MRA, mineralocorticoid receptor antagonist; NT-proBNP, N-terminal pro-brain natriuretic peptide.

Among the entire study cohort, 542 (64%) patients (age: 68, 57–77 years) were diagnosed with HF based on RHC, echocardiographic, and clinical data. Within this group, 160 patients (30%) had reduced EF (HFrEF, EF ≤ 40%), 73 (14%) had mid-range EF (HFmrEF, EF 41%–49%), and 309 (57%) had preserved EF (HFpEF, EF ≥ 50%). Additionally, 170 patients (age 66; 51–75 years) were diagnosed with PAH, either as new diagnosis based on the current RHC and echocardiography or as previously diagnosed patients on PAH treatment. Nineteen patients were diagnosed with constrictive pericarditis. Furthermore, RHC was performed in 51 cases post-heart transplantation, while 7 patients had left ventricular assist devices implanted. In 14 patients, chronic interstitial pulmonary disease had been diagnosed. Haemodynamic and echocardiographic evaluations were normal in 49 patients (5.7%). The haemodynamic characteristics are presented in *Table 2*.

**Table 2:** Haemodynamic measurements

***Q*_F_ (L/min)**	4.5 (3.6; 5.6)
***Qi*_F_ (L/min/m^2^)**	2.33 (1.92; 2.86)
***Q*_TD_ (L/min)**	4.3 (3.5; 5.4)
***Qi*_TD_ (L/min/m^2^)**	2.26 (1.87; 2.75)
**AVO_2_-difference (mL/L)**	53.5 (45.5; 63)
**SBP (mmHg)**	122 (105; 139)
**DBP (mmHg)**	71 (62; 80)
**PAWPm (mmHg)**	13 (9; 19)
**PAPm (mmHg)**	26 (20; 35)
**PAPs (mmHg)**	41 (31; 56)
**PAPd (mmHg)**	16 (11; 22)
**RAPm (mmHg)**	7 (4; 11)
**PVR_F_ (WU)**	2.42 (1.46; 3.92)
**PVR_TD_(WU)**	2.48 (1.58; 3.89)

*Q*_F_, cardiac output derived by direct Fick method; *Q*_TD_, cardiac output derived by thermodilution; *Qi*_F_, cardiac index derived by direct Fick method; *Qi*_TD_, cardiac index derived by thermodilution; AVO_2_-diff, arteriovenous difference; SBP, systolic blood pressure; DBP, diastolic blood pressure; PAWPm, Pulmonary artery capillary wedge; PAPm, mean pulmonary artery pressure; PAPs, systolic pulmonary artery pressure; PAPd, diastolic pulmonary artery pressure; RAPm, mean right atrial pressure; PVR_F_, pulmonary vascular resistance derived by direct Fick; PVR_TD_, pulmonary vascular resistance derived by thermodilution. Data are presented as median and lower and upper quartiles (*Q*1; *Q*3).

### Definitions and cohort overview

Both *Q*_F_ and *Q*_TD_ measurements were obtained in 852 patients. *Q*_F_ was 4.5 L/min (3.6–5.6), whereas *Q*_TD_ was 4.3 L/min (3.5–5.4; *P* < .001).

### Overall correlation

A strong linear association was observed between *Q*_F_ and *Q*_TD_ across the entire cohort (*r* = 0.79; *r*^2^ = 0.62; *P* < .001).

### Heart failure phenotypes

Within the subgroup of 542 patients with HF, the correlation persisted (*r* = 0.78) and did not differ significantly among HFpEF (*r* = 0.76), HFmrEF (*r* = 0.82), and HFrEF (*r* = 0.78). The median bias (Δ*Q*_TD–F_) ranged from −0.3 to −0.2 L/min across these phenotypes (*P* = .44).

### Rhythm and pulmonary arterial hypertension subgroups

Correlation coefficients were comparable between patients in sinus rhythm (*r* = 0.77; *n* = 306), atrial fibrillation (*r* = 0.78; *n* = 127), and paced rhythm (*r* = 0.77; *n* = 109). Similarly, in the PAH cohort (*n* = 170), the association was strong (*r* = 0.70; *P* < .001).

### Indexed flow strata

Indexed flow (*Qi*_F_) was stratified as low (≤2.2 L/min/m^2^), normal (2.2–4.1 L/min/m^2^), or high (>4.1 L/min/m^2^) (*Table 3*). Correlation between *Q*_TD_ and *Q*_F_ declined at high-flow: *r* = 0.63 in low-flow, *r* = 0.70 in normal-flow, and *r* = 0.45 in high-flow groups (all *P* < .001). Among patients with HF, those with low flow (*n* = 256) exhibited slightly weaker association (*r* = 0.63) compared with those with normal–high flow (*n* = 286; *r* = 0.73; *P* < .001).

**Table 3:** Comparison of thermodilution and Fick methods across cardiac index and tricuspid regurgitation subgroups

	Low *Qi* (*n* = 357)		Normal *Qi* (*n* = 461)		High *Qi* (*n* = 34)	
	TD	Fick	TD	Fick	TD	Fick
*Q* (L/min)	3.5 (2.9; 4.2)	3.4 (3.0; 4.0)**	4.9 (4.1; 5.7)	5.3 (4.5; 6.1)**	7.3 (5.6; 8.5)	9.3 (7.8; 10.3)**
*Qi* (L/min/m^2^)	1.8 (1.5; 2.2)	1.9 (1.6; 2.0)**	2.5 (2.2; 2.9)	2.7 (2.4; 3.2)**	3.8 (3.4; 4.6)	4.7 (4.3; 5.4)**

	TR-1 (*n* = 500)		TR-2 (*n* = 165)		TR-3 (*n* = 187)	
	TD	Fick	TD	Fick	TD	Fick
***Q* (L/min)**	4.8 (3.9; 5.7)	4.8 (4.0; 6.0)**	3.7 (3.1; 4.7)	4.0 (3.3; 4.9)*	4.0 (3.1; 4.7)	3.9 (3.2; 5.2)*
***Qi* (L/min/m^2^)**	2.4 (2.0; 2.9)	2.5 (2.1; 3.0)**	2.1 (1.7; 2.5)	2.1 (1.8; 2.6)*	2.1 (1.7; 2.5)	2.2 (1.68; 2.7)*

Upper panel: The median values of cardiac output (*Q*) and cardiac index (*Qi*) are presented for the three *Qi* subgroups: low (*Qi* ≤ 2.2 L/min/m^2^), normal (2.2 < *Qi* ≤ 4.1 L/min/m^2^), and high (*Qi* > 4.1 L/min/m^2^).

Lower panel: The median values of cardiac output (*Q*) and cardiac index (*Qi*) are shown for the three TR subgroups: mild TR (TR-1), moderate TR (TR-2), and severe TR (TR-3).

^*^ *P* < .05 between TD and Fick measurements.

^**^ *P* < .001.

### Pulmonary artery wedge pressure strata in heart failure

Among HF patients with normal–high *Qi*_F_, correlation remained consistent across PAWP strata: *r* = 0.73 at PAWP < 15 mmHg (*n* = 120) vs *r* = 0.74 at PAWP ≥ 15 mmHg (*n* = 166). Conversely, in the low-flow subgroup, correlation was stronger among those with elevated filling pressures [*r* = 0.67 at PAWP ≥ 15 mmHg (*n* = 169) vs *r* = 0.53 at PAWP < 15 mmHg (*n* = 87), *P* < .001; see Supplementary File and Table S1].

### Diagnostic performance for low flow

In ROC analysis, *Q*_TD_ demonstrated an AUC of 0.86 [standard error 0.013; 95% confidence interval (CI) 0.83–0.88; *P* < .001] for the detection of low flow (*Qi*_F_ < 2.2 L/min/m^2^). At the optimal *Q*_TD_ threshold of 2.2 L/min/m^2^, sensitivity was 78%, specificity 77%, PPV 71%, and NPV 83%. Among HFpEF, HFmrEF, and HFrEF patients, the prevalence of low flow was 40%, 49%, and 61%, respectively. Among these subgroups, the false-negative rate—i.e. patients with low *Qi*_F_ incorrectly classified as normal by *Q*_TD_ —was 25% in HFpEF, and 11% in both HFmrEF and HFrEF. The false-positive rate—patients with normal *Qi*_F_ incorrectly identified as low flow by *Q*_TD_—was 33% in HFpEF, 27% in HFmrEF, and 25% in HFrEF.

### Agreement

Bland–Altman plotting revealed a mean bias of −0.18 L/min (SD 1.04) and an overall APE of 13% (95% CI 5.8%–21.6%). *Q*_TD_ exceeded *Q*_F_ in 59% of low-flow, 31% of normal-flow, and 21% of high-flow cases.

Clinically significant disparity, defined as APE ≥ 20%, occurred in roughly 30% of both low- and normal-flow patients, but in over 50% of high-flow patients. Among those low- and normal-flow patients with APE ≥ 20%, *Q*_TD_ overestimated *Q*_F_ in 76% and 25% of cases, respectively. In contrast, of the high-flow patients with APE ≥ 20%, *Q*_F_ exceeded *Q*_TD_ in 89% of cases. By comparison, good agreement, defined as APE < 10%, was seen in ∼40% of low- and normal-flow patients, but in only 12% of high-flow patients.

**Figure 1:**
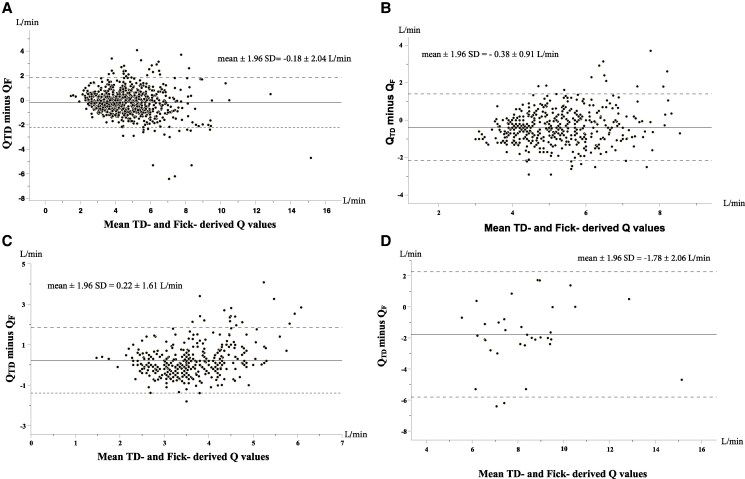
Bland–Altman plot demonstrating the agreement between thermodilution and direct Fick cardiac output (*Q*) measurements in the entire cohort (*A*), in the subgroup of patients with low cardiac index (*Qi* ≤ 2.2 L/min/m^2^) as measured by direct Fick (*B*), in the subgroup of patients with normal cardiac index (*Qi* = 2.2–4.1 L/min/m^2^) (*C*) and in the corresponding group with high cardiac index (*Qi* > 4.1 L/min/m^2^) (*D*)

**Figure 2:**
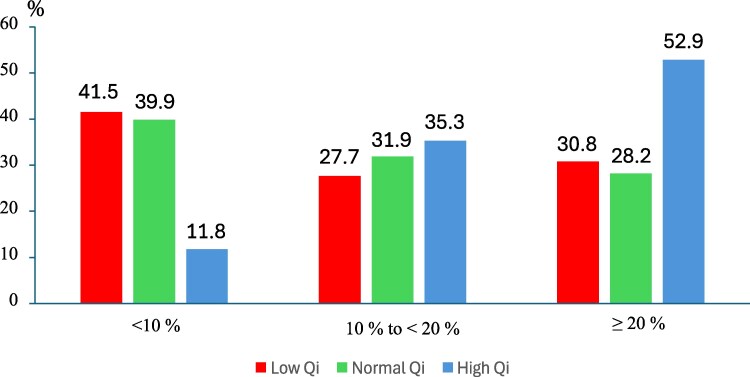
Prevalence of three categories of measurement error—<10%, 10 to <20%, and ≥20%—expressed as absolute per cent error [|(Δ*Q*_TD–F_/*Q*_F_) %|] between cardiac output values obtained by thermodilution (*Q*_TD_) and direct Fick (*Q*_F_). Data are presented as percentages of patients within each of the three categories of absolute per cent error for the low cardiac index (*Qi*) subgroup (red), the normal *Qi* subgroup (green), and the high *Qi* subgroup (blue)

*Figure 1A–D* illustrates the bias and limits of agreement, whereas *Figure 2* highlights the frequency of the various degrees of agreement thresholds.

### Tricuspid regurgitation effect on *Q*_TD_ and *Q*_F_ concordance

The impact of TR on the discrepancy *Q*_TD_ and *Q*_F_ was assessed across TR severity groups: mild (TR-1, *n* = 500; 58.7%), moderate (TR-2, *n* = 165; 19.4%), and severe (TR-3, *n* = 187; 21.9%) (*Table 3*). Correlation between *Q*_TD_ and *Q*_F_ was similar across groups (*r* = 0.75, 0.78, and 0.77, respectively). Δ*Q*_TD–F_ did not differ significantly [TR-1: −0.2 (−0.8, 0.3); TR-2: −0.17 (−0.6, 0.3); TR-3: −0.10 (−0.8, 0.4) L/min; *P* = .6], nor did the APE [TR-1: 13% (6, 21); TR-2: 12% (5, 21); TR-3: 15% (6, 24); *P* = .17].

### Multivariable analysis of factors associated with Δ*Q*_TD–F_

Linear regression analysis was performed to identify factors associated with the absolute difference between TD and Fick-derived *Q* (Δ*Q*_TD–F_). The model included two continuous variables (PAWPm, BSA) and three categorical variables: TR severity (mild–moderate–severe), *Qi*_F_ classification (low vs normal–high), and PH severity (non-PH, mild, moderate, severe). The model was statistically significant [*R*^2^ = 0.13; *F*(5,843) = 24; *P* < .001], explaining 18.0% of the variance in Δ*Q*_TD–F_. Low-flow status (*Qi*_F_ < 2.2 L/min/m^2^) was the strongest predictor (*β* = 0.34, *B* = 0.73; *P* < .001), followed by PAPm (*β* = 0.14; *P* < .001). Mean pulmonary artery wedge pressure was also significantly and inversely associated (*β* = −0.13; *P* < .001), while BSA and TR severity were not significant.

A second model using per cent difference (Δ*Q*_TD–F_%) showed similar results (*R*^2^ = 0.13; *P* < 0.001), with low *Qi*_F_, PAPm, and PAWP remaining significant predictors.

### Impact of *Q*_TD_–*Q*_F_ discrepancy on pulmonary hypertension classification

Among 608 patients with PH (71%), PAPm was 31 mmHg (25–39) and PVR_F_ 3 WU (2–4.9). *Q*_TD_–*Q*_F_ correlation was strong in PH-LHD (*r* = 0.83), non-PH (*r* = 0.72), and PAH (*r* = 0.70), all *P* < .001. Thermodilution consistently underestimated *Q*_F_ across PH severity, with bias declining from −0.3 L/min in non-PH to −0.1 L/min in moderate/severe PH (*P* < .05).

### Thermodilution’s classification performance for pulmonary vascular resistance ≥ 2 WU

PVR_TD_ ≥2 WU accurately identified elevated PVR as defined by the reference Fick method (PVR_F_ ≥2 WU; prevalence 74%), with AUC = 0.97, sensitivity 97%, specificity 85%, PPV 95%, NPV 90%, and accuracy 94%. Diagnostic performance was stable across flow states (sensitivity/specificity: 96%/87% in low flow; 98%/84% in normal–high flow).

A prespecified multivariable logistic regression model was constructed to distinguish cases in which PVR_F_ ≥2 WU but PVR_TD_ <2 WU (false negatives) from those in with PVR_F_ < 2 WU but PVR_TD_ ≥ 2 WU (false positives). The model demonstrated an AUC of 0.82 (95% CI 0.78–0.86). Covariates included BSA, AVO_2_-diff, TR grade, PAWPm, age, sex, and PAPm; only AVO_2_-diff (*P* = .045) and PAWPm (*P* = .055) independently predicted the differentiation between false negatives and false positives.

### Thermodilution’s classification performance for pulmonary vascular resistance ≥ 5 WU

For identifying markedly elevated PVR, defined by the reference standard Fick-derived measurements (PVR_F_ ≥5 WU; prevalence 24%), TD demonstrated a sensitivity of 90%, specificity of 96%, PPV of 87%, and NPV of 97%.

In a logistic regression including BSA, AVO_2_-diff, TR grade, mean PAWP, age, gender, and PAPm category, only BSA (*P* = .046) and age (*P* = .041) independently predicted the differentiation between false negatives and false positives at the PVR ≥ 5 WU threshold. All other covariates were non-significant (Supplementary Table S2A and B).

### Thermodilution’s diagnostic performance for pulmonary vascular resistance ≥2 WU by heart failure phenotype

We further analysed the diagnostic accuracy of TD-derived PVR against the reference Fick-derived method (PVR_F_ ≥2 WU) across HF phenotypes in patients with PH (Supplementary Table S3). Sensitivity remained high in all groups (HFrEF: 98%, HFmrEF: 97%, HFpEF: 96%), supporting the reliability of TD for detecting elevated PVR. Specificity varied, with HFmrEF demonstrating the lowest value (64%) compared with HFrEF (84%) and HFpEF (86%). The PPV was highest in HFpEF (95%), followed by HFrEF (93%), and lowest in HFmrEF (85%).

## Discussion

In this retrospective comparison of direct Fick vs TD *Q* measurements, three principal observations emerged. First, although *Q*_F_ and *Q*_TD_ demonstrated strong correlation, one-third of paired assessments diverged by ≥20%. Second, TD systematically underestimated *Q* in normal-flow states but overestimated it under low-flow conditions. Third, while TD exhibited only moderate sensitivity for reduced cardiac index, it maintained excellent discrimination for precapillary involvement in PH.

Our findings corroborate earlier observations of TD’s moderate accuracy for assessing *Q* and its tendency to overestimate in low-output states. Narang *et al*.^13^ reported discrepancies exceeding 25% between TD and Fick measurements in one-third of cases, aligning with our finding of a >20% intermodality difference in ∼30% of cases. Similarly, both studies reveal a persistent discrepancy between the two methods across low- and normal-flow conditions, though the nature of the discrepancy varied depending on the flow state.^13^ In support of this, a smaller investigation documented systematic TD overestimation in 16 patients with low *Q*, partially aligning with our finding of higher TD measurements in about two-thirds of low-output cases.^14^ In contrast, Hillis *et al*. reported relatively high agreement between methods, with <10% difference in 64% of low-*Q* and 71% of normal-*Q* cases. By comparison, only 40% of our paired assessments fell within that range in both the normal and the low-*Q* groups. This discrepancy may be attributable to our larger sample size and a higher proportion of low-*Q* cases (42% vs 15% in Hillis’ study).^12^ Hoeper *et al*.^5^ similarly noted minimal average bias between *Q*_TD_ and *Q*_F_ in patients with PAH, but still reported ∼30% intermodality variance.^5^ In our larger PAH cohort (*n* = 170), *Q*_TD_ overestimated *Q*_F_ in low-output states by an average of 15% ± 33%, and slightly underestimated it in normal flow (−1.3% ± 19.6%). Differences in baseline haemodynamics—particularly higher mean pulmonary artery pressures and lower *Q* in the Hoeper cohort—may partly explain the observed variation. Our results reveal that while *Q*_TD_ and *Q*_F_ remained well correlated across HF subtypes, TD was less reliable in HFpEF, with a 25% false-negative rate for detecting low flow—more than double that of HFrEF and HFmrEF (both 11%). The risk of false positives was also highest in HFpEF (33%), highlighting the potential for both under- and overestimation of flow in this HF group.

Notwithstanding the potential sources of error inherent to Fick measurements, the overestimation of *Q* by TD in hypokinetic states is consistent with the underlying physical principles of this modality. Chronic low-output states often involve enlargement of right-sided cardiac chambers and an increase in endocardial surface area, both of which enhance heat dissipation and thereby reduce the TD curve, leading to inflated *Q* estimates. Experimental evidence by Hayes *et al*.^20^ in animal models supports this mechanism, demonstrating that local thermal conduction affects *Q*_TD_ readings based on sampling site.

To our knowledge, this is the first study to systematically examine the impact of measurement discrepancies in *Q* on the diagnostic performance of PVR in PH. We found that TD-derived PVR values demonstrated high diagnostic accuracy against the reference Fick-derived measurements. At the recommended diagnostic threshold of 2 WU, TD demonstrated strong rule-out performance and even stronger rule-in capability for elevated PVR, despite variability in agreement with Fick-derived *Q* and their differential behaviour across flow states. Importantly, this diagnostic robustness extended across HF subtypes. Negative predictive value remained high across all subgroups (>87%), supporting TD’s value in excluding elevated PVR regardless of EF category.

Overall, the false-negative rate was low (3.3%), while the false-positive rate (∼15%) raises concerns for overdiagnosis of a precapillary PH component, especially in patients with normal flow. Nevertheless, false-positive cases generally showed only mildly elevated PVR_TD_ with the corresponding PVR_F_ values near the upper limit of normal, underscoring the need for caution when interpreting borderline TD-based elevations in PVR.

Thermodilution’s diagnostic performance remained strong even at the prognostically relevant threshold of ≥5 WU. With a high NPV and low false-positive rate, TD reliably excluded markedly elevated PVR. However, the PPV of 87% and false-negative rate of roughly 10% indicates a non-negligible risk of underdiagnosing clinically significant PVR elevation, which might limit its utility for confirmatory purposes. Given the tendency of TD to overestimate *Q* in low-flow states—common among patients with significant precapillary involvement—our findings indicate that direct Fick measurement should be favoured particularly in HF patients undergoing evaluation for transplantation.

Finally, our findings demonstrate that severe TR exerts no significant impact on agreement between TD and Fick for *Q*, despite theoretical concerns regarding right ventricular remodelling and altered injectate transit. Early investigations yielded discordant results,^16,18,21–23^ yet in our substantially larger cohort, TR severity, including severe, massive, and torrential, neither correlation strength nor bias magnitude differed significantly across TR strata.

In summary, TD demonstrates reduced accuracy in low-flow conditions but retains strong diagnostic performance for detecting elevated PVR. These findings support the clinical utility of TD in routine haemodynamic assessment, while highlighting the need for careful interpretation in cases of borderline PVR elevation and in high-risk populations such as transplant candidates.

### Limitations

A major limitation of the current study is its retrospective nature. Measurements of $\dot{V}O_{2}$ were performed by different operators, which may introduce variability in the direct Fick assessments. However, our institution adheres to a standardized protocol for RHC, and all operators undergo extensive training prior to working in the catheterization lab. Additionally, the Fick method is routinely applied in our lab, with considerable in-house expertise. Data on pleural effusion were not systematically available and thus could not be analysed, although, according to institutional protocol, all RHC were performed in haemodynamically stable patients, which minimizes the likelihood of major fluid shifts that might confound TD measurements. Although this study spans a 10-year period, no methodological changes occurred during this time in either the TD or direct Fick techniques. Moreover, our study cohort is the largest reported in this area, suggesting that any occasional measurement errors would likely have a minimal impact on the overall findings.

## Supplementary Material

xvaf001_Supplementary_Data

## Declarations

### Data Availability

The data underlying this article will be shared on reasonable request to the corresponding author.

### Pre-registered Clinical Trial Number

None supplied.

## References

[1] D’Alonzo GE, Barst RJ, Ayres SM, Bergofsky EH, Brundage BH, Detre KM, et al Survival in patients with primary pulmonary hypertension: results from a national prospective registry. Ann Intern Med 1991;115:343–9. 10.7326/0003-4819-115-5-343 1863023

[2] Humbert M, Kovacs G, Hoeper MM, Badagliacca R, Berger RMF, Brida M, et al 2022 ESC/ERS guidelines for the diagnosis and treatment of pulmonary hypertension. Eur Respir J 2023;61:2200879. 10.1183/13993003.00879-2022 36028254

[3] Singh M . Pulmonary hypertension: an update of Dx and Tx guidelines. J Fam Pract 2023;72:72–83. 10.12788/jfp.0561 36947786

[4] Ganz W, Donoso R, Marcus HS, Forrester JS, Swan HJC. A new technique for measurement of cardiac output by thermodilution in man. Am J Cardiol 1971;27:392–6. 10.1016/0002-9149(71)90436-X 4929422

[5] Hoeper MM, Maier R, Tongers J, Niedermeyer J, Hohlfeld JM, Hamm M, et al Determination of cardiac output by the Fick method, thermodilution, and acetylene rebreathing in pulmonary hypertension. Am J Respir Crit Care Med 1999;160:535–41. 10.1164/ajrccm.160.2.9811062 10430725

[6] Baeza H, Chamorro G, Lanas F, Escobar E. [Measurement of cardiac output by thermodilution. Correlation with the direct Fick method (author’s transl)]. Rev Med Chil 1978;106:784–7. 741122

[7] Branthwaite MA, Bradley RD. Measurement of cardiac output by thermal dilution in man. J Appl Physiol 1968;24:434–8. 10.1152/jappl.1968.24.3.434 5640735

[8] Enghoff E, Michaëlsson M, Pavek K, Sjögren S. A comparison between the thermal dilution method and the direct Fick and the dye dilution methods for cardiac output measurements in man. Acta Soc Med Ups 1970;75:157–70. 4941433

[9] Hodges M, Downs JB, Mitchell LA. Thermodilution and Fick cardiac index determinations following cardiac surgery. Crit Care Med 1975;3:182–4. 10.1097/00003246-197509000-00002 765060

[10] Levine BA, Sirinek KR. Cardiac output determination by thermodilution technique: the method of choice in low flow states. Proc Soc Exp Biol Med 1981;167:279–83. 10.3181/00379727-167-41164 7232436

[11] Kubo SH, Burchenal JE, Cody RJ. Comparison of direct Fick and thermodilution cardiac output techniques at high flow rates. Am J Cardiol 1987;59:384–6. 10.1016/0002-9149(87)90829-0 3812301

[12] Hillis LD, Firth BG, Winniford MD. Analysis of factors affecting the variability of Fick versus indicator dilution measurements of cardiac output. Am J Cardiol 1985;56:764–8. 10.1016/0002-9149(85)91132-4 3904383

[13] Narang N, Thibodeau JT, Parker WF, Grodin JL, Garg S, Tedford RJ, et al Comparison of accuracy of estimation of cardiac output by thermodilution versus the Fick method using measured oxygen uptake. Am J Cardiol 2022;176:58–65. 10.1016/j.amjcard.2022.04.026 35613956 PMC9648100

[14] van Grondelle A, Ditchey RV, Groves BM, Wagner WW, Reeves JT. Thermodilution method overestimates low cardiac output in humans. Am J Physiol 1983;245:H690–2. 10.1152/ajpheart.1983.245.4.H690 6624939

[15] Nishikawa T, Dohi S. Errors in the measurement of cardiac output by thermodilution. Can J Anaesth 1993;40:142–53. 10.1007/BF03011312 8443853

[16] Cigarroa RG, Lange RA, Williams RH, Bedotto JB, Hillis LD. Underestimation of cardiac output by thermodilution in patients with tricuspid regurgitation. Am J Med 1989;86:417–20. 10.1016/0002-9343(89)90339-2 2648822

[17] Heerdt PM, Pond CG, Blessios GA, Rosenbloom M. Inaccuracy of cardiac output by thermodilution during acute tricuspid regurgitation. Ann Thorac Surg 1992;53:706–8. 10.1016/0003-4975(92)90342-2 1554289

[18] Konishi T, Nakamura Y, Morii I, Himura Y, Kumada T, Kawai C. Comparison of thermodilution and Fick methods for measurement of cardiac output in tricuspid regurgitation. Am J Cardiol 1992;70:538–9. 10.1016/0002-9149(92)91205-I 1642196

[19] Hahn RT, Zamorano JL. The need for a new tricuspid regurgitation grading scheme. Eur Heart J Cardiovasc Imaging 2017;18:1342–3. 10.1093/ehjci/jex139 28977455

[20] Hayes BE, Will JA, Zarnstorff WC, Bisgard GE. Limitations of thermodilution cardiac output measurements in the rat. Am J Physiol 1984;246:H754–60. 10.1152/ajpheart.1984.246.6.H754 6742141

[21] Khirfan G, Ahmed MK, Almaaitah S, Almoushref A, Agmy GM, Dweik RA, et al Comparison of different methods to estimate cardiac index in pulmonary arterial hypertension. Circulation 2019;140:705–7. 10.1161/CIRCULATIONAHA.119.041614 31424987 PMC7182442

[22] Gonzalez J, Delafosse C, Fartoukh M, Capderou A, Straus C, Zelter M, et al Comparison of bedside measurement of cardiac output with the thermodilution method and the Fick method in mechanically ventilated patients. Crit Care 2003;7:171–8. 10.1186/cc1848 12720564 PMC270608

[23] Hamilton MA, Stevenson LW, Woo M, Child JS, Tillisch JH. Effect of tricuspid regurgitation on the reliability of the thermodilution cardiac output technique in congestive heart failure. Am J Cardiol 1989;64:945–8. 10.1016/0002-9149(89)90851-5 2801567

