# Supplementary material for: Agreement of thermodilution and direct Fick methods for cardiac output across varying haemodynamic conditions
Source: ESC Heart Fail. 2026 Jan 8;13(1):xvaf001. doi: 10.1093/eschf/xvaf001 (PMC12974568; doi:10.1093/eschf/xvaf001)
Supplement: xvaf001_Supplementary_Data [file xvaf001_supplementary_data.zip › Supplementary file.docx]

**Supplementary file**

**Methods:**

Estimated *Q* based on Fick principle was also calculated by using VO₂ approximated from BSA. BSA was calculated using the Du Bois formula: BSA=0.007184×height (cm)^0.725^×weight (kg)^0.425^. VO_2_ was subsequently estimated as:

VO_2_​est=125×BSA (mL/min). The estimated Fick-derived *Q* was then computed as: *Q*_Fest_=VO_2_est/AVO_2_-diff.

**Results**:

The analysis of the relative percent difference between *Q_TD_* and *Q_F_* revealed a systematic tendency for *Q_TD_* to overestimate *Q_F_* values, particularly at lower *Qi_F_* levels. As depicted in Figure S1A-D (see Supplementary Material), this overestimation was consistently observed across the three TR groups, with the effect being most pronounced in TR-3.

In HF group, correlations within PAWP strata were rather consistent for each *Q*i subgroup as shown in the table S1 (see Supplementary Material).

**Correlation of *Q*_TD_, *Q*_F_ Measurements in LVAD patients**

In the subgroup of the patients on LVAD (n= 7) the correlation between *Q*_TD_ and *Q*_F_ was excellent r=0.91, p=0.016 with Δ*Q*_TD-F_=0.1 (-0.72;0.75) L/min.

**Correlation of *Q*_TD_, *Q*_F_ and *Q*_Fest_ Measurements**

A robust association was observed among the three *Q* methods in the overall cohort. *Q*_F_ correlated strongly with *Q*_TD_ (r = 0.79, p < 0.001) and *Q*_Fest_ (r = 0.77, p < 0.001), while *Q*_TD_ also showed a high correlation with *Q*_Fest_ (r = 0.79, p < 0.001). In the subgroup with normal-high flow (*Qi*_F_ >2.2 L/min/m²; n = 495), *Q*_F_ remained strongly correlated with both *Q*_TD_ (r = 0.74, p < 0.001) and *Q*_Fest_ (r = 0.71, p < 0.001), and a similarly strong correlation was observed between *Q*_TD_ and *Q*_Fest_ (r = 0.72, p < 0.001). Among patients with low-flow (*Qi*_F_ < 2.2 L/min/m²; n = 357), all pairwise correlations persisted but were attenuated: *Q*_F_ and *Q*_TD_ (r = 0.6), *Q*_F_ and *Q*_Fest_ (r = 0.63), and *Q*_TD_ and *Q*_Fest_ (r = 0.75), all p < 0.001.
